# An enzyme-like imprinted-polymer reactor with segregated quantum confinements for a tandem catalyst†

**DOI:** 10.1039/c7ra12320e

**Published:** 2018-01-04

**Authors:** Wenjing Wei, Tingting Zhou, Shuping Wu, Xiaojuan Shen, Maiyong Zhu, Songjun Li

**Affiliations:** Institute of Polymer Materials, School of Materials Science & Engineering, Jiangsu University Zhenjiang 212013 China Lsjchem@ujs.edu.cn http://material.ujs.edu.cn/info/1151/1026.htm

## Abstract

This study was aimed at addressing the present challenge in tandem catalysts, as to how to furnish catalysts with tandem catalytic-ability without involving the precise control and man-made isolation of different types of catalytic sites. This objective was realized by constructing an enzyme-like imprinted-polymer reactor made of a unique polymer composite inspired from the compartmentalization of cells, a composite of a reactive imprinted polymer (containing acidic catalytic sites), and encapsulated metal nanoparticles (acting as catalytic reduction sites). The compilation of two types of catalytic sites with admissible access allowed this reactor to behave like compartments of cells for enzymatic reactions and hence catalytically constituted two quantum interaction-segregated domains, which led to the occurrence of catalytic tandem processes. Unlike the reported functional reactors that run tandem catalysis by largely depending on the precise control and man-made isolation of different types of catalytic sites, tandem catalysis in this reactor run naturally with segregated quantum confinements, which does not involve the precise control and isolation of different types of catalytic sites. This protocol presents new opportunities for the development of functional catalysts for complicated chemical processes.

## Abstract 2

This study was aimed at addressing the present challenge in tandem catalysts: how to furnish catalysts with tandem catalytic-ability without involving the precise control and man-made isolation of different types of catalytic sites.

## Graphical abstract

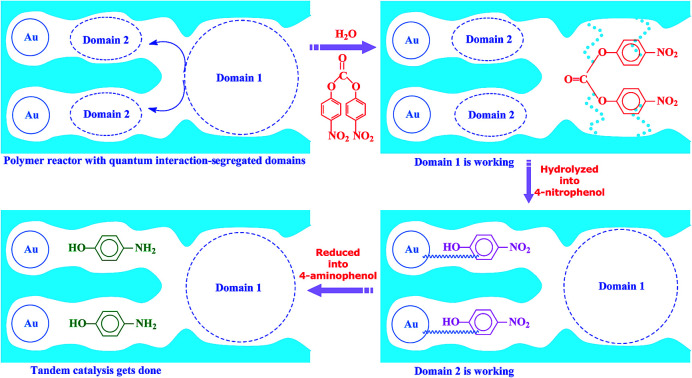

## 1. Introduction

The significance of tandem catalysts for modern chemical industries is beyond doubt because of less number of synthesis, separation, and purification steps for these catalysts and requirement of much less solvents. The use of tandem catalysts in chemical syntheses would allow consecutive reactions to proceed in a concurrent and harmonious fashion, endowing the system with one-pot synthetic-ability. Prominent among these catalysts are functional reactors,^1–3^ composites of different types of catalytic sites. Since the earliest endeavors, exemplified by acidic and basic-site-containing architectures,^4,5^ functional reactors have exhibited tandem catalytic-ability. This outcome is the result of their unique bi-functional catalytic properties, in which the acidic sites allow one reaction and the basic sites are responsible for another coupled reaction. In this way, the use of functional reactors leads to the occurrence of tandem catalytic-ability. However, practical applications of functional reactors have been unremarkable over the years; one important reason behind this is the fact that tandem catalysis at functional reactors essentially relies on the precise control and man-made isolation of different types of catalytic sites^6–8^ to avoid undesired side reactions, cross reactions, and even incompatible reactions. It is, therefore, unrealistic to adopt these functional reactors for practical applications due to the high requirement of structural textures. As such, the actual tandem catalysis would require functional reactors that can be easily prepared (ideally without involving the precise control and man-made isolation of different types of catalytic sites), allow some specified reactions, and avoid undesired reactions. Unfortunately, it is confoundedly difficult (if not impossible) to directly acquire these functional reactors based on the currently available results.

For centuries, mankind has been learning and acquiring knowledge from nature. A body of knowledge is now available. Researchers have learnt how to create and resolve complicated issues from the inspirations sought from nature; one such inspiration is the cells,^9,10^ which share a promising prospect with struggling functional reactors with regard to their tandem and specified catalytic-ability. During the course of evolution, the cells in biosystems have been confronted with similar challenges and needed one-pot processes to synthesize the desired products from the available primary molecules *via* adverse-reaction-free multi-step reactions. Millions of years of evolution has enabled them to realize this objective by dividing into compartments for main enzymatic reactions,^11,12^ where the desired multi-step reactions are allowed to run in the individual compartments in a streamlined way, and almost no accommodation is available for undesired molecules due to the specificity of the enzymatic reactions. No extra control of different types of catalytic sites is necessary due to the enzymes that act as a streamlined scaffold for the occurrence of tandem catalysis. Although the exact mechanism remains to be understood, clues to these catalytically segregated compartments lie in microscopic quantum mechanics,^13,14^ in which the interactions between different types of catalytic sites (enzymes) and substrates are the fundamental reason behind tandem catalysis. The tandem and specified catalytic-abilities would normally involve admissible access and consecutive catalytic behaviors in these compartments where the former allows accommodations for desired reactions and the latter is responsible for the catalytic tandem processes. In this way, the cells, as whole reactors, provide the tandem and specified catalytic-abilities.

**Scheme 1:**
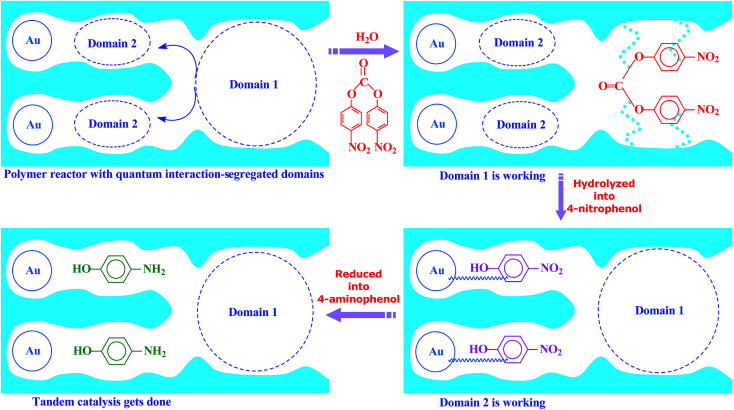
The proposed mechanism for the enzyme-like imprinted-polymer reactor with segregated quantum confinements with respect to tandem catalysis.

Inspired by this delicate principle in nature, we aimed at addressing the present challenge in functional reactors by constructing an enzyme-like imprinted-polymer reactor made of a unique polymer composite inspired from cell compartmentalization, a composite of a reactive imprinted polymer (containing acidic catalytic sites), and encapsulated Au nanoparticles (acting as catalytic reduction sites) (named MIP–Au–NP–BNPC, Scheme 1). The compilation of two types of catalytic sites with admissible access allowed this reactor to behave like cell compartments for enzymatic reactions, and hence catalytically constituted two quantum interaction-segregated domains with each responsible for one admissible reaction (the detailed theories for the microscopic quantum mechanics are shown in the ESI† material). The molecular recognition properties at the reactive imprinted polymer allowed access to the desired molecules, whereas the compilation of two types of catalytic sites may be responsible for the tandem hydrolysis and catalytic reduction. No extra control and man-made isolation of different types of catalytic sites were necessary since the tandem catalytic-ability was naturally formed with segregated quantum confinements in the enzyme-like polymer composite. In this way, this reactor demonstrated tandem catalytic-ability. In this regard, two well-coupled substrates, *i.e.*, bis(4-nitrophenyl) carbonate (BNPC) and 4-nitrophenol (NP), capable of catering to the acidic catalytic sites and reduction sites in this reactor were selected as the tentative templates and catalytic substrates for the fabrication of the imprinted polymer.^15,16^ The catalytic hydrolysis of the former (*i.e.*, BNPC) would lead to the formation of the latter (*i.e.*, NP), which in the presence of admissible access and catalytic metal nanoparticles can be further reduced to 4-aminophonel (AP). To highlight the essence of the segregated quantum confinements on the catalytic tandem process, this polymer reactor has been henceforth discussed in conjunction with the corresponding control reactors prepared with either of the two templates (*i.e.*, BNPC and NP) and without any template (*i.e.*, remaining with either one or no domain in these control reactors; named MIP–Au–BNPC, MIP–Au–NP, and NIP–Au, respectively) (herein, MIP means the molecularly imprinted polymer reactor prepared with either of the two templates (*i.e.*, BNPC and NP) and NIP means the non-imprinted-polymer reactor that is prepared without any template). The objective of this study was to demonstrate that functional reactors, acting as tandem catalysts with segregated quantum confinements, can be prepared by this protocol and hence provide opportunities for the development of functional catalysts for complicated chemical processes.

## 2. Interpretation of quantum mechanics on the natural formation of tandem catalytic-ability

The detailed description of the microscopic quantum mechanics of this polymer reactor is shown in the ESI† material. To show the essence of the segregated quantum confinements on the catalytic tandem process, this polymer reactor has been henceforth discussed together with the corresponding control reactors MIP–Au–BNPC, MIP–Au–NP, and NIP–Au, in which the interactions of the substrate molecules with the two imprints (*i.e.*, BNPC (*m*_1_) and NP (*m*_2_)-imprinted networks) are considered two tandem potential-wells. The corresponding Schrödinger equation for the whole tandem system is as follows:1
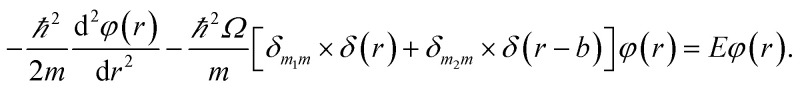


Herein, the Kronecker deltas (*δ*_*mm*′_; either 1 or 0) and Dirac functions (*δ*(*r*); either 0 or ∞) show the different mechanic-behaviors between MIP–Au–NP–BNPC and the control reactors. Kronecker deltas are used because these prepared reactors either contain (*δ*_*mm*_ = 1) or lack (*δ*_*mm*′_ = 0) the corresponding imprints in their polymeric networks. The imprint-containing reactors would have strong interactions with the corresponding substrate molecules (*i.e.*, the imprinted molecules) (*V* ∼ −∞), whereas the imprint-lacking reactors would not involve any essential interactions with the engaging substrate molecules (*V* ∼ 0). The solution to eqn (1) is2
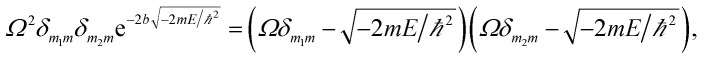
and provides allowed-energies for the natural formation of tandem catalytic-ability. A zero-value in either of the Kronecker deltas would lead to *E* ≡ 0 and hence result in a failure of the catalytic tandem system (as apparent in the case for these control reactors, which lack necessary imprints in the polymeric networks). The tandem catalysis at MIP–Au–NP–BNPC becomes feasible with3
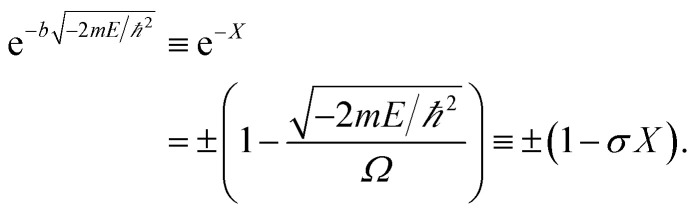


The allowed energies can be achieved from the transcendental equation by plotting e^−*X*^ and ±(1 − *σX*) on the same grid. The two intersection-points showed pre-requisites for the natural formation of tandem catalytic-ability in this reactor (*i.e.*, segregated quantum confinements necessary for the natural formation of tandem catalytic-ability)4
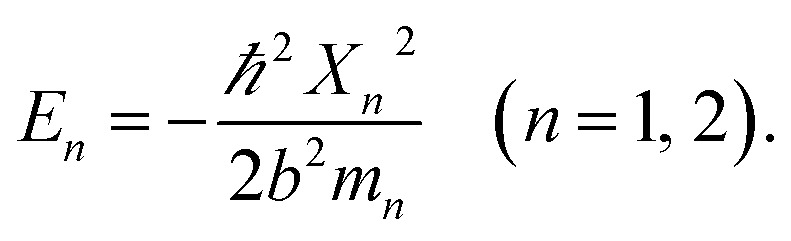


The segregated quantum confinements in this reactor catalytically constitute two quantum interaction-segregated domains, which lead to the occurrence of tandem catalysis. Hence, once the initial substrate molecules are mounted in this reactor, the substrate molecules would be subjected to the corresponding quantum confinements in the two domains in which each is responsible for one admissible reaction. As such, differing from the reported functional reactors that run tandem catalysis by largely depending on the precise control and man-made isolation of different types of catalytic sites, the tandem catalysis at this reactor runs naturally with the segregated quantum confinements, which does not involve the precise control and man-made isolation of different types of catalytic sites. In this way, this reactor demonstrated tandem catalytic-ability.

## 3. Experimental

### 3.1. Preparation of polymer reactors

**Scheme 2:**
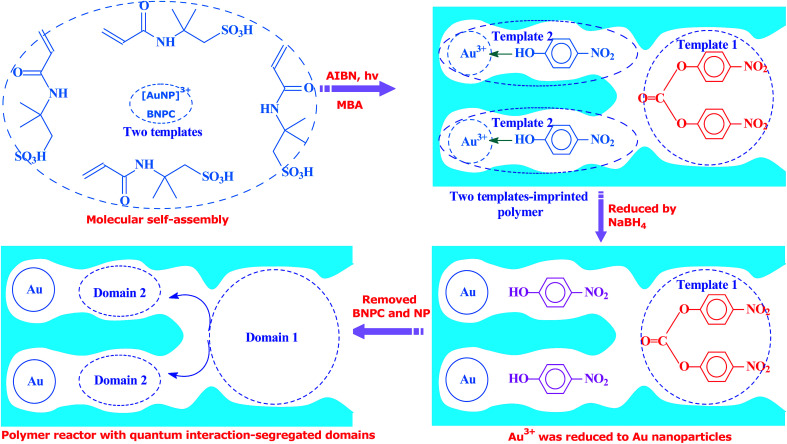
Schematic for the preparation of polymer reactors.

Unless otherwise noted, the chemicals used herein were of analytic grade and used as received from Sigma-Aldrich. The polymer reactor, as outlined in Scheme 1, was prepared using the classic molecular imprinting technology^17^ (Scheme 2). The templates used for the imprinting process were BNPC (30.4 mg; 0.1 mmol) and the complex of [Au(NP)_2_]^3+^, which was formed by adding chloroauric acid (78.8 mg; 0.2 mmol) to NP-dimethylsulfoxide solution (0.4 mmol mL^−1^; 5 mL).^18,19^ The functional monomer (2-acrylamido-2-methylpropanesulfonic acid; 0.21 g, 1.0 mmol), crosslinker (*N*,*N*′-methylene bisacrylamide (MBA); 0.54 g, 3.5 mmol), and the initiator (AIBN; 0.2 g) were added to the template solution. After being dispersed and degassed *via* sonication and nitrogen, the mixture system was placed under ultraviolet radiation (365 nm; 24 h) for complete polymerization and formation of the imprinted polymer precursor. The encapsulated Au ions were then reduced with an excess of sodium borohydride (ten-fold with regard to the amount of Au ions; 2 h). The formed polymer composite was profusely washed with ethanol containing 10% acetic acid to remove the imprinted BNPC and NP moieties. The resulting polymer reactor was then dried in nitrogen and ground into a size of ∼60 mesh for further applications.

Considering the whole catalytic process in which Au was necessary for the tandem catalysis, three control reactors (*i.e.*, MIP–Au–BNPC, MIP–Au–NP, and NIP–Au) containing Au were also prepared under comparable conditions. For these control reactors, either one or no template was used for the fabrication of imprinted species; this hence allowed these control reactors to have either one or no domain in their polymer networks.

### 3.2. Characterization

The TEM images of the prepared reactors were obtained using a JEM-2100 transmission electron microscope (TEM) (Japan). The surface morphology was observed using a JSM-7800F scanning electron microscope (SEM) (Japan). The BET surface-area and porosity were investigated using a NOVA2000 nitrogen analyzer (USA). The FTIR spectra were obtained using a Nicolet MX-1E apparatus (USA). The absorption bands of the surface plasma resonance (SPR) were obtained using a Lambda 25 UV spectrophotometer (USA). The energy-dispersive spectra (EDS) were obtained using a MIRA3-XMU apparatus (USA).

### 3.3. Temperature-programmed desorption

Temperature-programmed desorption (TPD) was used to evaluate the interaction between the prepared reactors and substrates to identify the molecular recognition properties in the two domains.^20,21^ The molecular recognition properties in the corresponding domains would permit a larger retention as compared to the case of the control reactors that are short of the domains. As such, the prepared reactors (0.2 mg) that were pre-adsorbed with 10 μL substrates (0.05 μmol mL^−1^ acetonitrile) were added to online U-shaped quartz tubes (4 mm at I.D.) in a TPD device composed of a gas chromatograph equipped with a thermal conductivity detector and a data processing system. The quartz tubes were then heated under flowing nitrogen (60 mL min^−1^; 0.45 MPa) at 10 °C min^−1^ from room temperature up to the temperature at which the absorbed substrates desorbed. The desorbing signal was obtained by the data processing system.

### 3.4. Catalytic properties

The catalytic properties of the prepared reactors were evaluated using batch formats.^22^ Considering that the catalytic tandem process was made of two consecutive steps, the catalytic properties were tested in both the presence and absence of sodium borohydride (as a reducer) to better understand the individual and the consecutive catalytic processes. The initial concentration of BNPC was 0.2 μmol mL^−1^ (10 mL PBS; pH 7.0) (NaBH_4_ if present, four-fold in contrast to BNPC). The content of these reactors used in every testing was 1.0 mg mL^−1^. The catalytic behavior was spectrophotometrically monitored against time (scanning span: 15 min) using a UV-2700 spectrophotometer (Japan), and the catalytic activities were determined from the average of three runs. Due to the potential effect of spontaneous reactions on the catalytic process, the reactions of BNPC in the absence of any reactor were also performed under comparable conditions, and accordingly, the effect was neglected from the overall activities of these reactors.

### 3.5. Desorbing electrochemistry

Desorbing electrochemistry was further used to investigate the interaction between the prepared reactors and substrates to further acquire information on the two domains.^23,24^ Using an electrochemical workstation equipped with a conventional three-electrode configuration (a Au-plate working electrode, a Pt-wire counter electrode, and a Ag/AgCl reference electrode), the prepared reactors (10 mg) that pre-absorbed with ∼2 μmol templates were placed in the electrochemical cells encircled by a diffusion-eliminating sonication apparatus (supporting electrolyte: 10 mL PBS; pH 7.0). The desorbing behavior of the absorbed substrates was monitored by circularly scanning the system until stable desorption profiles were reached (scanning range, −0.3 ∼ −1 V; scanning rate, 1 mV s^−1^).

## 4. Results and discussion

### 4.1. FTIR, TEM, SEM, EDS, and SPR analyses

**Fig. 1:**
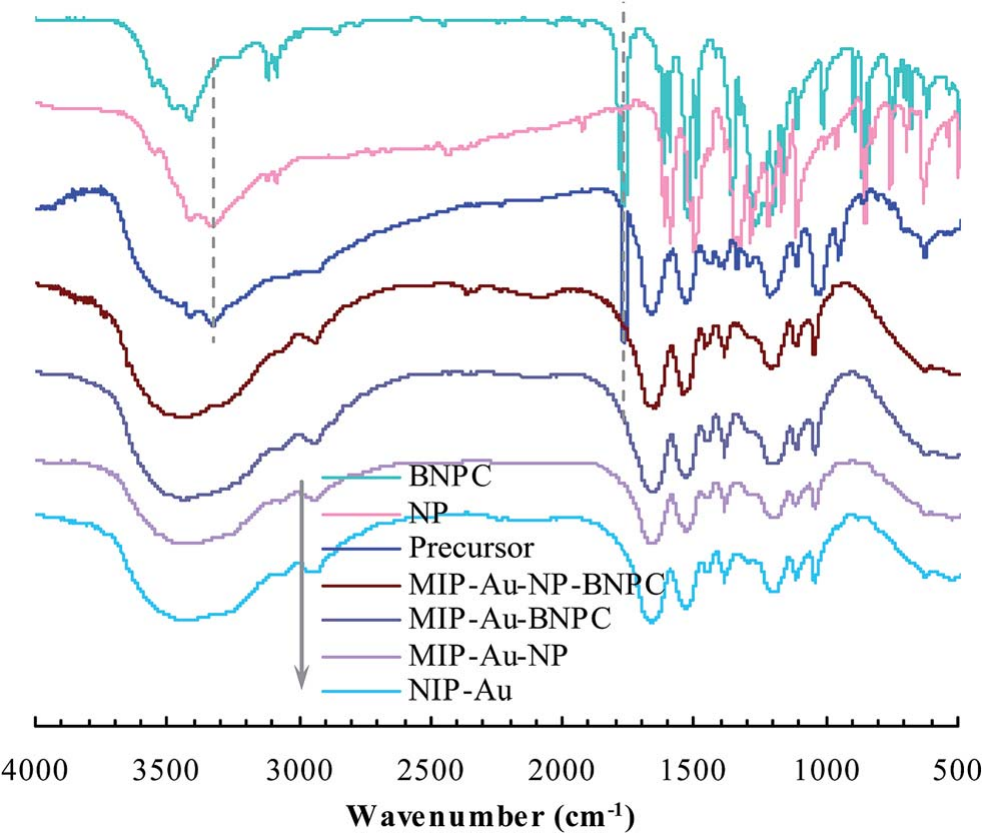
FTIR spectra of the prepared polymer reactors.

As aforementioned, the polymer reactor MIP–Au–NP–BNPC was prepared using BNPC and NP as templates. The encapsulated Au ions were reduced with an excess of sodium borohydride. The imprinted BNPC and NP moieties were then removed from the polymeric matrix; this resulted in the formation of imprinted species (Scheme 2). FTIR spectroscopic analysis was first used to monitor the imprinting process, as shown in Fig. 1. Herein, four major bands (3000–3750, 1600–1800, 1300–1550, and 1000–1300 cm^−1^) appeared in the spectrum of this reactor. These bands were composite due to the multi-component composition of the reactor, spectroscopically corresponding to the stretching of O–H/N–H, C=O, S=O, and C–N/C–C bonds.^25,26^ For addressing these absorption bands, the FTIR spectra of the three control reactors (*i.e.*, MIP–Au–BNPC, MIP–Au–NP, and NIP–Au) and two templates (*i.e.*, BNPC and NP), along with the MIP–Au–NP–BNPC precursor (in which the imprinted BNPC and NP moieties had not been removed from the polymeric matrix of MIP–Au–NP–BNPC), are also shown in Fig. 1. The MIP–Au–NP–BNPC precursor included the major bands of BNPC and NP at ∼3400 and 1750 cm^−1^, respectively. After removing BNPC and NP moieties from the precursor, the spectrum of the resulting reactor (*i.e.*, MIP–Au–NP–BNPC) became comparable to the spectra of other reactors. In conjunction with the preparation process (Scheme 2), this outcome implied the occurrence of imprinting behaviors and hence the generation of the imprints in the imprinted MIP–Au–NP–BNPC system (further discussion on the specific interaction is presented in Section 4.2).

**Fig. 2:**
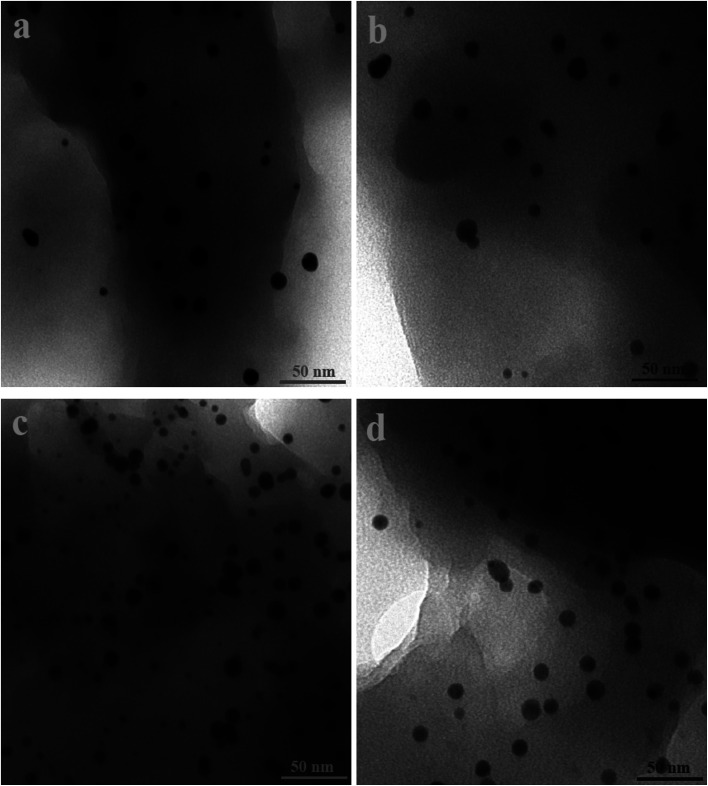
TEM images of the metal nanoparticles encapsulated in the prepared reactors (a) MIP–Au–NP–BNPC; (b) MIP–Au–BNPC; (c) MIP–Au–NP; and (d) NIP–Au.

**Fig. 3:**
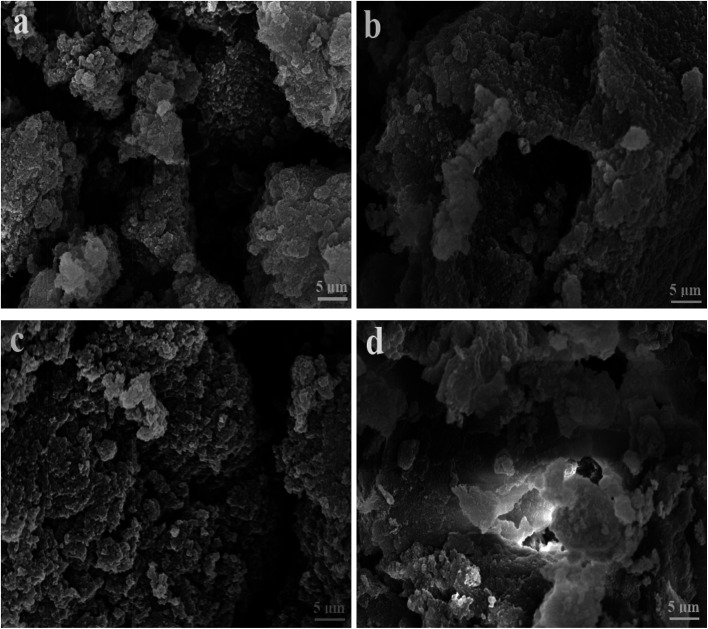
SEM images of the prepared reactors (a) MIP–Au–NP–BNPC; (b) MIP–Au–BNPC; (c) MIP–Au–NP; and (d) NIP–Au.

**Fig. 4:**
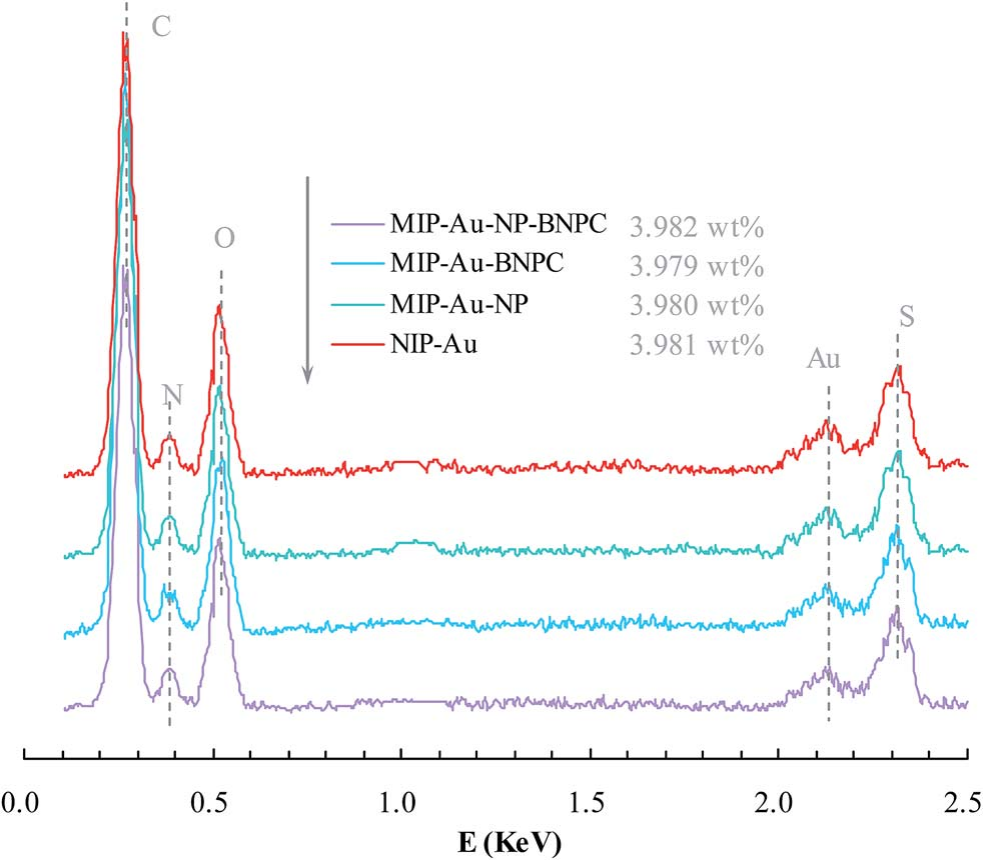
EDS spectra of the prepared reactors.

**Fig. 5:**
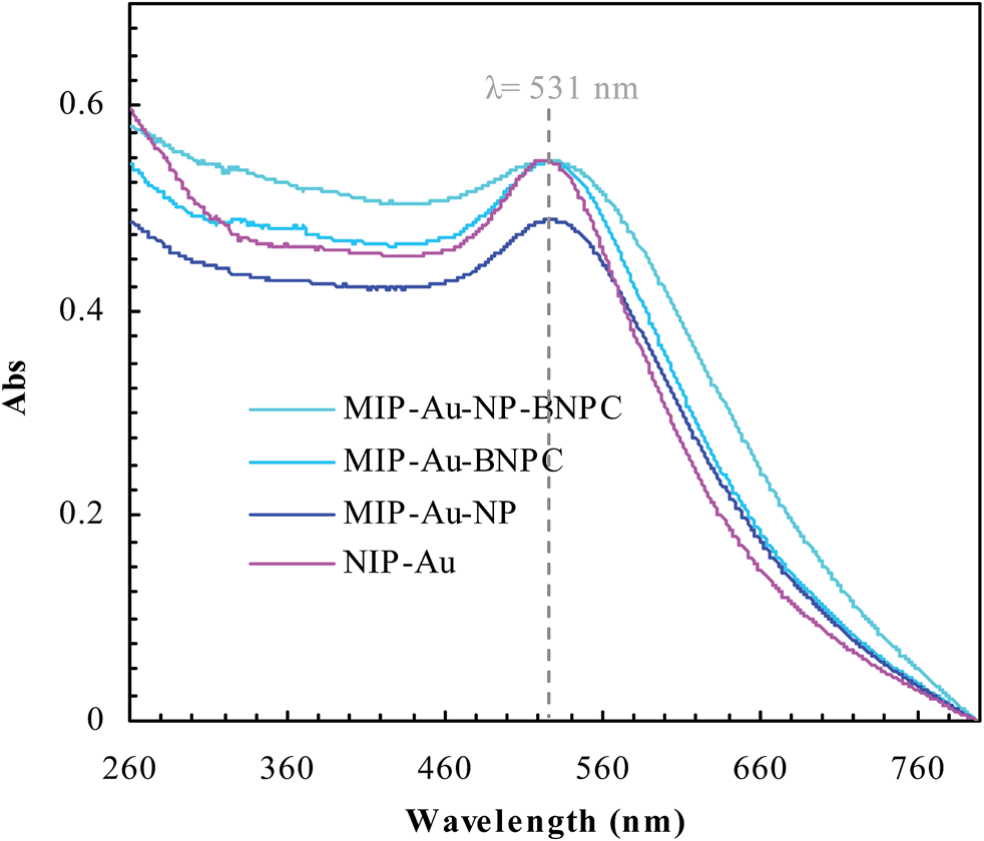
SPR spectra of the prepared reactors.

**Fig. 6:**
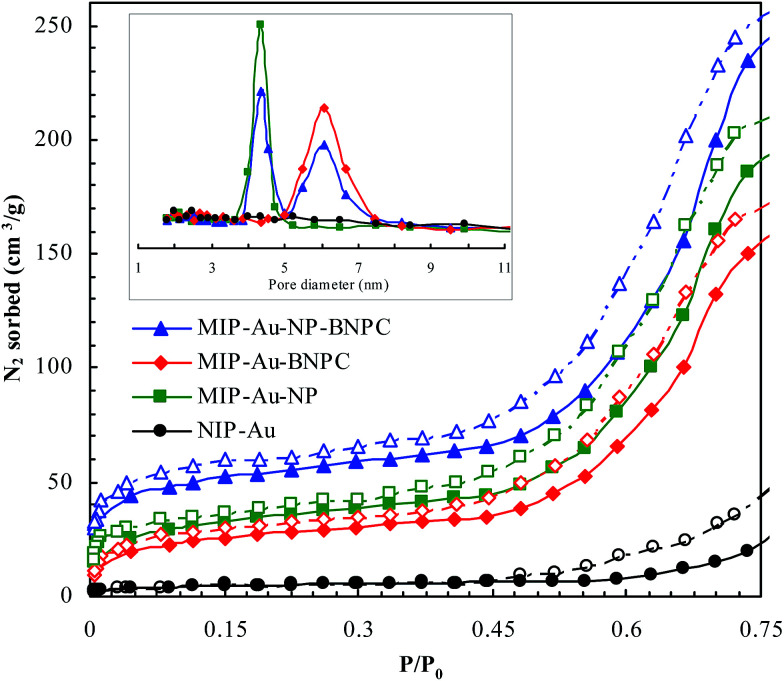
BET sorption isotherms of these prepared reactors (in which the closed symbols represented nitrogen adsorption and the open symbols represented desorption; there was no hysteresis loop available in the non-imprinted NIP–Au in contrast to these imprinted reactors that had the hysteresis loops under *P*/*P*_0_ < 0.35).

Fig. 2 and 3 show the SEM and TEM images, displaying the morphology of both the polymeric carriers and the metal nanoparticles encapsulated in these carriers. Au nanoparticles with the size of ∼15 nm were encapsulated in these polymer carriers. The presence of Au nanoparticles was further evidenced by the EDS and SPR spectra (typically at 520–540 nm),^27^ and accordingly, the Au loadings achieved were ∼4.0 wt% (Fig. 4 and 5). Fig. 6 shows the BET sorption isotherms of these reactors. MIP–Au–NP–BNPC, MIP–Au–BNPC, MIP–Au–NP, and NIP–Au exhibited, respectively, two, one, one, and no microspore distributions (<10 nm),^28^ in good agreement with the preparation of these reactors in which two, one, one, and no templates were used. Compared with NIP–Au, the other reactors had a larger surface area and higher pore volume (Table 1). These larger values may be related to the contribution of the templates during the preparation process that normally affects the resulting polymer reactors. Hence, these polymer reactors were prepared in the desired form.

**Table 1:** BET analysis of the prepared reactors

Prepared reactor	Surface area (m^2^ g^−1^)	Pore volume (μL g^−1^)
MIP–Au–NP–BNPC	142.7	63.9
MIP–Au–BNPC	108.2	59.8
MIP–Au–NP	114.6	61.2
NIP–Au	21.9	48.3

### 4.2. Specific interaction between the reactors and the substrate

**Fig. 7:**
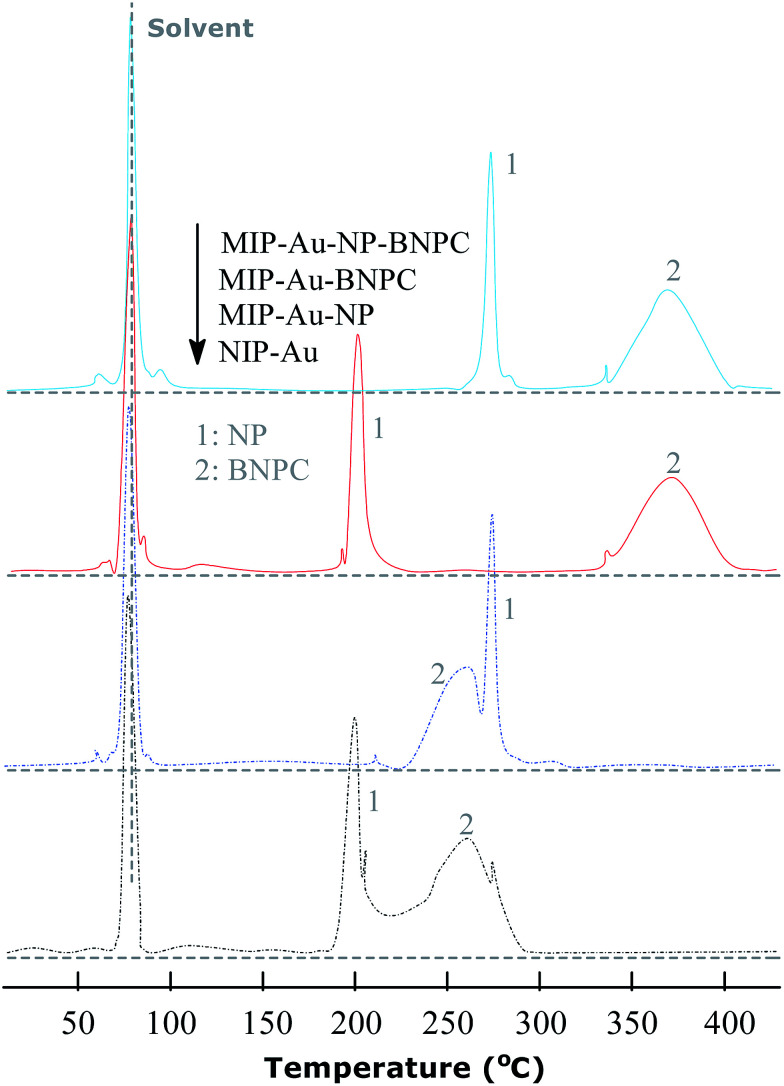
TPD profiles for the desorption of NP and BNPC from the prepared reactors.

The TPD experiments were further performed to address the interaction between the prepared reactors and substrates. A relatively stronger interaction with the substrates would enable the reactors to have larger retention as compared to a relatively weaker interaction. As shown in Fig. 7, the template molecule NP, which desorbed from MIP–Au–NP–BNPC, MIP–Au–BNPC, MIP–Au–NP, and NIP–Au, appeared at 268, 201, 270, and 199 °C, respectively. The NP-imprinted MIP–Au–NP–BNPC and MIP–Au–NP exhibited stronger interactions with NP as compared to the NP-lacking NIP–Au and MIP–Au–BNPC. The outcome was comparable in the case of BNPC in which the BNPC-imprinted MIP–Au–NP–BNPC and MIP–Au–BNPC also exhibited stronger interactions with BNPC (367 and 368 °C, respectively), as compared to the case of BNPC-lacking MIP–Au–NP and NIP–Au (262 and 260 °C, respectively). In conjunction with the studies discussed in Section 4.1, these outcomes again reflected the consequence of molecular imprinting. Since molecular recognition by the imprinted polymers was essentially a result of the induced molecular memory (*i.e.*, the imprints of the template molecules), stronger interactions at the corresponding imprinted reactors were expected.

As further shown in Fig. 7, both the NP-imprinted MIP–Au–NP–BNPC and MIP–Au–NP exhibited comparable desorption temperatures for NP (268 and 270 °C, respectively). The BNPC-imprinted MIP–Au–NP–BNPC and MIP–Au–BNPC also exhibited comparable desorption temperatures for BNPC (367 and 368 °C, respectively). In contrast, the non-imprinted NIP–Au behaved like either MIP–Au–BNPC for NP or MIP–Au–NP for BNPC, with respect to the desorption temperatures (*i.e.*, NIP–Au showed almost the same desorption temperatures as the reactors prepared without using the corresponding templates). This outcome strongly suggested that the imprints of template-molecules were capable of accommodating the corresponding substrate molecules, and only the substrates marched could gain access to the corresponding domains. As explained in the preparation process (Scheme 2), MIP–Au–NP–BNPC was prepared using both NP and BNPC templates, whereas other reactors were achieved using either one or no template. This would allow MIP–Au–NP–BNPC to have two corresponding domains in the polymer networks as compared to the control reactors that have either one or no domain in the polymer networks. In this way, these reactors due to the domains led to the corresponding retentions in the TPD profiles.

### 4.3. Catalysis and tandem processes

**Fig. 8:**
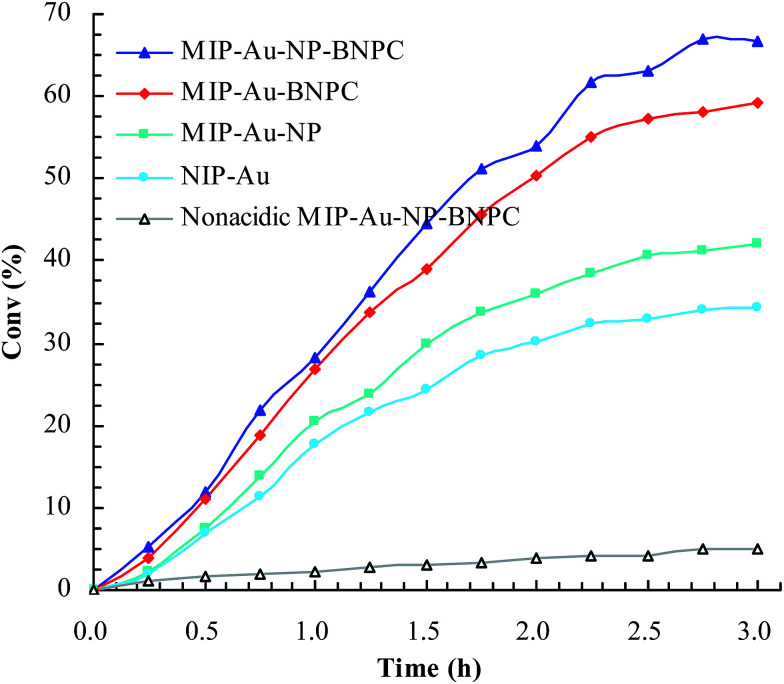
Catalytic activities for the hydrolysis of BNPC at the prepared reactors.

**Fig. 9:**
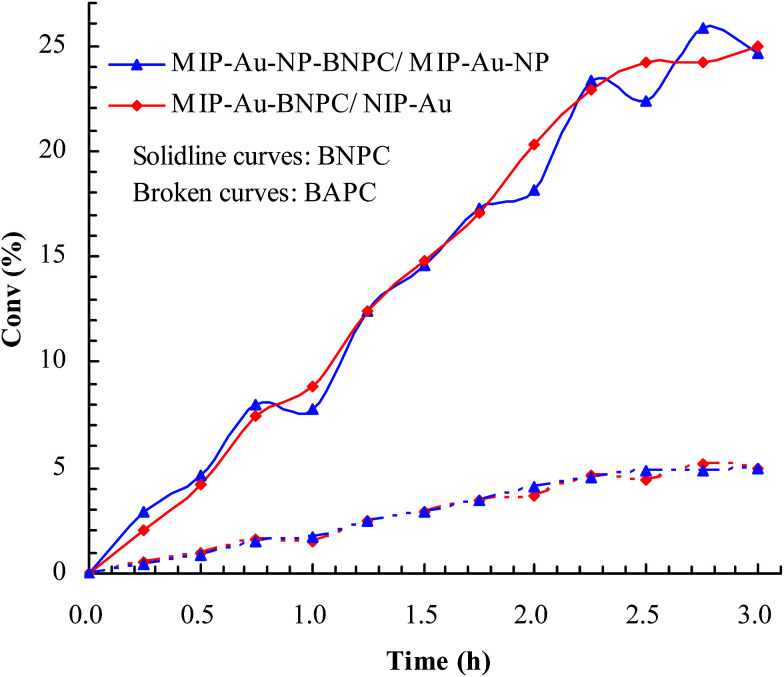
Contribution of the BNPC-imprinted species in MIP–Au–NP–BNPC and MIP–Au–BNPC to the selective catalysis.

Considering that the catalytic tandem process consisted of two consecutive steps, as aforementioned, the catalytic activities were hence first tested in the absence of sodium borohydride to have a glimpse into the catalysis in one individual step (such as the catalytic hydrolysis) (Fig. 8). For a comparative purpose, we also included the hydrolysis of BNPC at a nonacidic MIP–Au–NP–BNPC reactor prepared by replacing acidic monomers with neutral acrylamide, as shown in Fig. 8. All the acidic-site-containing reactors showed much higher catalytic activities in contrast to the nonacidic reactor. The acidic sites acted as a catalyst for the hydrolysis of BNPC. It was noted that the BNPC-imprinted MIP–Au–NP–BNPC and MIP–Au–BNPC demonstrated higher catalytic activities as compared to other reactors prepared without using BNPC. The BNPC-imprinted species evidently played a role in the hydrolysis of BNPC. After the catalytic activities of MIP–Au–NP–BNPC and MIP–Au–BNPC were correspondingly deducted by MIP–Au–NP and NIP–Au, the contribution of the imprints of BNPC to the hydrolysis was hence exposed (Fig. 9). Interestingly, the contributions of the imprints of BNPC to the hydrolysis in both MIP–Au–NP–BNPC and MIP–Au–BNPC were nearly identical. This outcome, when coupled with the surface areas that were not proportional to the hydrolytic activities, implied that the imprints of BNPC in MIP–Au–NP–BNPC and MIP–Au–BNPC acted as an independent domain for accommodating the hydrolysis of BNPC. For addressing this, guaiacol carbonate (GAC), as the analogue of BNPC, was further selected as the control. The contributions of the imprints of BNPC in MIP–Au–NP–BNPC and MIP–Au–BNPC to the hydrolysis of the analogue were also nearly identical. Furthermore, both MIP–Au–NP–BNPC and MIP–Au–BNPC demonstrated a catalytic preference for BNPC than for GAC. The effect remained consistent with time (Fig. 9). These outcomes strongly suggested that catalysis by the imprinted reactors, by virtue of the imprints, was an accommodation-regulated process. In conjunction with the TPD profiles, we can expect that the tandem catalysis of BNPC at MIP–Au–NP–BNPC would become feasible in the presence of sodium borohydride. The imprinted acidic-sites allowed accommodations for the hydrolysis of BNPC, whereas the encapsulated Au nanoparticles in the presence of admissible access would take responsibility for the following reduction. In this way, this reactor demonstrated the tandem catalytic-ability.

**Fig. 10:**
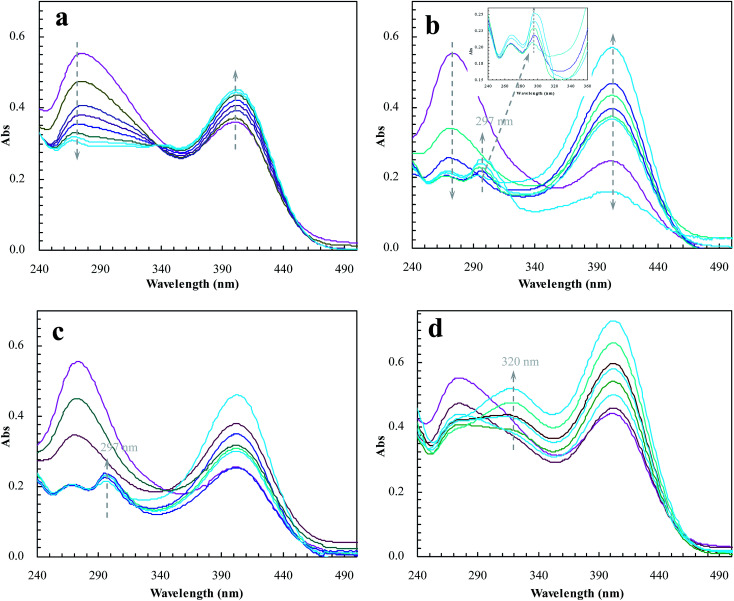
Changes in the UV spectrum of BNPC in the presence of sodium borohydride at the prepared reactors (a) NIP–Au; (b) MIP–Au–NP–BNPC; (c) MIP–Au–NP; and (d) MIP–Au–BNPC.

To meet the expectation, UV spectroscopic analysis was used to monitor the catalytic process of BNPC in the presence of sodium borohydride. The catalytic process of BNPC at NIP–Au led to a decreasing peak for BNPC (272 nm) and an increasing peak for NP (400 nm) (Fig. 10a), exhibiting the straightforward hydrolysis from BNPC to NP. There was no catalytic tandem process occurring at NIP–Au due to the lack of access to the encapsulated Au nanoparticles. In contrast, the catalytic process of BNPC at MIP–Au–NP–BNPC was more complicated and led to a decreasing peak for BNPC (272 nm) and nonetheless an increasing peak for AP (297 nm) (Fig. 10b). The formed NP intermediate in the presence of admissible access was further reduced to AP at MIP–Au–NP–BNPC. The desired catalytic tandem process took place at MIP–Au–NP–BNPC.

The result was similar at MIP–Au–NP (Fig. 10c); the AP peak was nonetheless a little smaller than that achieved at MIP–Au–NP–BNPC because of the less amount of NP released from the hydrolysis of BNPC. It was worth noting that MIP–Au–BNPC further led to the formation of bis(4-aminophenol) carbonate (BAPC; 320 nm) except for the case of NP (Fig. 10d); this might be related to the straightforward reduction of BNPC to BAPC. The BNPC-imprinted species played a role in the straightforward reduction of BNPC. The imprinted reactors, as already explained, were capable of accommodating the corresponding substrate molecules, and only the substrates marched could gain access to the corresponding domains. As such, the imprints of BNPC at MIP–Au–BNPC admitted access for BNPC with respect to the encapsulated Au nanoparticles; this, therefore, led to the straightforward reduction of BNPC. It was hence clear from these outcomes that the catalytic tandem process became feasible only under the pre-requisite that the catalytic sites were compiled with necessary accommodations. As described in the quantum mechanics, the compilation of the two-type catalytic sites with admissible accommodations allowed this reactor to behave like the cell compartments for enzymatic reactions, which catalytically constituted two quantum interaction-segregated domains and hence led to the occurrence of catalytic tandem processes. Further tests on the catalytic reproducibility indicated that the reactivity of MIP–Au–NP–BNPC would not be subject to significant decrease after a series of catalytic cycles, and this reactor was hence relatively stable. As such, this reactor led to potential opportunities for the development of functional catalysts for complicated chemical processes.

### 4.4. Desorption electrochemistry

**Scheme 3:**
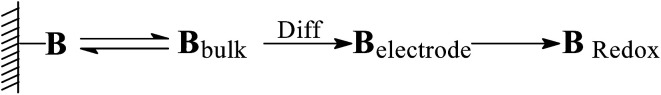
Schematic of the desorption electrochemical process with the binding molecule B.

**Fig. 11:**
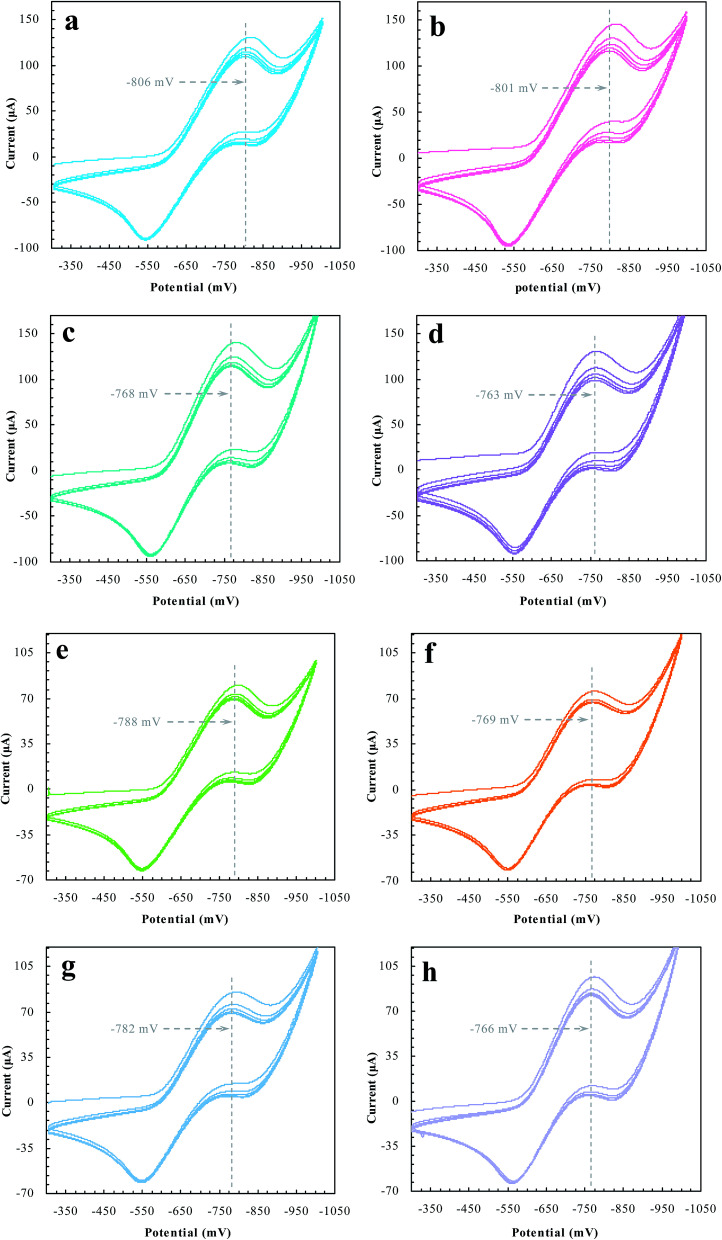
The desorption-reduction profiles of BNPC (a–d) and NP (e–h) at the prepared reactors (a) and (e) MIP–Au–NP–BNPC; (b) and (f) MIP–Au–BNPC; (c) and (g) MIP–Au–NP; (d) and (f) NIP–Au.

Desorption electrochemistry was further used to investigate the interaction between the prepared reactors and substrates.^23,24^ It has been known that the potential to reduce/oxidize a binding molecule depends upon the binding strength. Stronger binding needed relatively more energy to overcome the binding, hence resulting in a larger redox potential. The theory and details, as outlined in Scheme 3, have been described elsewhere.^29,30^ Despite the reactions, the substrate in the electrochemical system would normally involve desorption, diffusion to the surface of electrodes, and terminal redox processes. Once the diffusion was eliminated with sonication, the desorption behavior of the substrate was, therefore, directly correlated to the change of the redox potential. As such, the desorption electrochemistry provided valuable information on the two domains. As shown in Fig. 11, BNPC, which was attached to MIP–Au–NP–BNPC, MIP–Au–BNPC, MIP–Au–NP, and NIP–Au, showed the desorption-reduction peaks at −806, −801, −768, and −763 mV, respectively (a, b, c, and d). The BNPC-imprinted MIP–Au–NP–BNPC and MIP–Au–BNPC exhibited stronger interactions with BNPC as compared to the BNPC-lacking NIP–Au and MIP–Au–NP. The outcome was similar in the case of NP where the NP-imprinted MIP–Au–NP–BNPC and MIP–Au–NP also exhibited stronger interactions with NP (e and g) as compared to the cases of NP-lacking MIP–Au–BNPC and NIP–Au (f and h). When combined with both the TPD profiles and catalytic studies, these outcomes again reflected the consequence of the two domains in MIP–Au–NP–BNPC, capable of accommodating the corresponding substrates. Reminiscent of the quantum-mechanic theories, the two domains in MIP–Au–NP–BNPC allowed this reactor to naturally function with segregated quantum confinements, which hence led to the occurrence of tandem catalytic-ability.

## 5. Conclusions

This study was aimed at addressing the present challenge in tandem catalysts by constructing an enzyme-like imprinted-polymer reactor made of a unique polymer composite inspired from cell compartmentalization, a composite of a reactive imprinted polymer (containing acidic catalytic sites), and encapsulated metal nanoparticles (acting as catalytic reduction sites). The compilation of two types of catalytic sites with admissible access allowed this reactor to behave like cell compartments for enzymatic reactions, and hence catalytically constituted two quantum interaction-segregated domains with each responsible for one admissible reaction. The imprinted acidic-sites provided accommodations for the hydrolysis of the imprinted molecules, whereas the encapsulated Au nanoparticles in the presence of admissible access would take responsibility for the following reduction. No extra control and man-made isolation of different types of catalytic sites were necessary since the tandem catalytic-ability at this reactor was naturally formed with the segregation of microscopic quantum mechanics in the enzyme-like polymer composite. Differing from the reported functional reactors that often run tandem catalysis by depending on the precise control and man-made isolation of different types of catalytic sites, the tandem catalysis at this reactor runs naturally with the segregated quantum confinements, which does not involve the precise control and isolation of different types of catalytic sites. The study of this reactor hence allowed opportunities for developing functional catalysts for complicated chemical processes. The future development in this field will significantly increase the potential for applications and lead to the appearance of novel catalytic materials and functional catalysts.

## Conflicts of interest

There are no conflict of interest to declare.

## Supplementary Material

RA-008-C7RA12320E-s001
