# Supplementary material for: An enzyme-like imprinted-polymer reactor with segregated quantum confinements for a tandem catalyst
Source: RSC Adv. 2018 Jan 4;8(3):1610–20. doi: 10.1039/c7ra12320e (PMC9077128; doi:10.1039/c7ra12320e)
Supplement: RA-008-C7RA12320E-s001 [file RA-008-C7RA12320E-s001.pdf]

Electronic Supplementary Material (ESI) for RSC Advances.
This journal is © The Royal Society of Chemistry 2017

Supplementary Materials

For the manuscript entitled "Enzyme-like imprinted-polymer reactor with segregated quantum confinements for tandem catalyst"

Interpretation of quantum mechanics on the natural formation of tandem catalytic-ability

The basic theory for describing microscopic particles and their motion subject to a potential field has been addressed by the Schrödinger equation:[1]

$$\left[-\frac{\hbar^2}{2m}\nabla^2+V(r)\right]\varphi(r)=E\varphi(r) \quad (\textit{Time independent}), \text{ or}$$

$$i\hbar\frac{\partial}{\partial t}\Psi(r,t)=\left[-\frac{\hbar^2}{2m}\nabla^2+V(r)\right]\Psi(r,t) \quad (\textit{Time dependent}). \qquad (1)$$

Here i is the imaginary unit, $\hbar$ is the reduced Planck constant (1.05457×10^{-34} J s), φ (or Ψ) is the wave function of the quantum system (relating to position r and/or the time t), E is the total energy of the quantum state φ, and V is the potential energy. For the convenience of mathematic handling, the time-independent equation is in common use. Once the time-independent equation is solved, the general solution of the time-dependent equation is simple and straightforward by multiplying by the exponential function exp($-iEt/\hbar$).[2]

For the actual applications, some simplified models have to be adopted given the diversity of particlar motions and the complication of actual potential fields. The quantum-mechanic behaviors in catalysis are even more complicated because of the composition of multiple components and the multiplicity of catalytic sites. Nonetheless, the quantum-mechanic behaviors in the catalytic tandem systems can be delicately simplified, given the fact that the occurrence of tandem catalysis originates normally from two different-type catalytic sites in which one is responsible for the precursor reaction and another one is responsible for the following reaction.[3-5] Once both of the catalytic sites are regulated with admissible access, the admitted reactions are hence allowed to take place. As in the present case, the coupled pair bis(4-nitrophenyl) carbonate (BNPC) and 4-nitrophenol (NP) were used as templates (*i.e.*, as the imprinted molecules) to fabricate the MIP-Au-NP-BNPC reactor, which hence admitted access for BNPC and NP. The initial hydrolysis of the

former (*i.e.*, BNPC) would lead to the formation of the latter (*i.e.*, NP) and which in the presence of admissible access and catalytic metal nanoparticles can be further reduced into 4-aminophonel (AP). In contrast, either one or not-any template had been used for the preparation of these control reactors, which only allowed these control reactors to remain with either mono- or not any access in the polymeric networks. As such, there was no catalytic tandem reaction normally available in these control reactors due to the lack of necessary accommodations. As such, one simplified model for the quantum-mechanic behaviors in these prepared reactors can be suggested as **Scheme S1**, where the Kronecker deltas ($\delta_{mm'}$; either 1 or 0) and Dirac functions ($\delta(r)$; either 0 or ∞) are used to summarize the different mechanic-behaviors between MIP-Au-NP-BNPC and these control reactors [6]

$$V(r) = -\frac{\hbar^2\Omega}{m}[\delta_{m_1m}\cdot\delta(r)+\delta_{m_2m}\cdot\delta(r-b)]. \qquad (2)$$

Herein, the interactions of the substrate molecules with two different "imprints" (*i.e.*, BNPC (m_1) and NP (m_2)-imprinted networks) are considered two tandem potential-wells (Ω shows the "size" of the potential wells). The use of Kronecker deltas lies in the fact that these reactors either contain (δ_{mm}=1) or lack ($\delta_{mm'}$=0) the corresponding 'imprints' in the polymeric networks. The 'imprints'-containing reactors would have strong interactions with the corresponding substrate molecules (*i.e.*, the imprinted molecules) (V~ -∞), whereas the 'imprints'-lacking reactors would not involve essential interactions with the engaging substrate molecules (V~ 0). As such, the Schrödinger equation for the whole tandem system would read

$$-\frac{\hbar^2}{2m}\frac{d^2\varphi(r)}{dr^2}-\frac{\hbar^2\Omega}{m}[\delta_{m_1m}\cdot\delta(r)+\delta_{m_2m}\cdot\delta(r-b)]\varphi(r)=E\varphi(r) \text{ or}$$

$$\frac{d^2\varphi(r)}{dr^2}-k^2\varphi(r)=-2\Omega[\delta_{m_1m}\cdot\delta(r)+\delta_{m_2m}\cdot\delta(r-b)]\varphi(r), \qquad (3)$$

where $k\equiv\sqrt{-2mE/\hbar^2}$ (the bound motion of the substrate molecules in these reactors requires the energy E< 0). To solve Eqn (3), the Fourier-transformation method has to be adopted given the mathematic complication. The Fourier transformation of Eqn (3) shows

$$
\begin{aligned}
-\omega^2\psi(\omega) - k^2\psi(\omega) &= -2\Omega\int_{-\infty}^{+\infty}[\delta_{m_1m}\cdot\delta(r) + \delta_{m_2m}\cdot\delta(r-b)]\varphi(r)e^{-i\omega r}dr \\
&= -2\Omega[\delta_{m_1m}\varphi(0) + \delta_{m_2m}\varphi(b)e^{-iwb}]
\end{aligned}, \qquad (4)
$$

and hence reads

$$
\psi(\omega) = \frac{2\Omega[\delta_{m_1m}\varphi(0) + \delta_{m_2m}\varphi(b)e^{-iwb}]}{k^2 + \omega^2}, \qquad (5)
$$

where $\psi(\omega) = \int_{-\infty}^{+\infty}\varphi(r)e^{-i\omega r}dr$ (*i.e.*, the Fourier transformation of $\varphi(r)$). The inverse transformation of Eqn (5) shows the wave function of the substrate molecules in the tandem system as follows

$$
\varphi(r) = \frac{\Omega}{k}[\delta_{m_1m}\varphi(0)e^{-k|r|} + \delta_{m_2m}\varphi(b)e^{-k|r-b|}]. \qquad (6)
$$

As in Eqn (6), $\varphi(r)$ at r=0 and r=b say

$$
\begin{aligned}
\varphi(0) &= \frac{\Omega}{k}[\delta_{m_1m}\varphi(0) + \delta_{m_2m}\varphi(b)e^{-kb}] \\
\varphi(b) &= \frac{\Omega}{k}[\delta_{m_1m}\varphi(0)e^{-kb} + \delta_{m_2m}\varphi(b)]
\end{aligned}, \qquad (7)
$$

and hence read

$$
\begin{aligned}
-\Omega\delta_{m_2m}e^{-kb}\varphi(b) &= (\Omega\delta_{m_1m} - k)\varphi(0) \\
(\Omega\delta_{m_2m} - k)\varphi(b) &= -\Omega\delta_{m_1m}e^{-kb}\varphi(0)
\end{aligned}. \qquad (8)
$$

Dividing one relationship by another one in Eqn (8) would give

$$
\Omega^2\delta_{m_1m}\delta_{m_2m}e^{-2kb} = (\Omega\delta_{m_1m} - k)(\Omega\delta_{m_2m} - k). \qquad (9)
$$

Eqn (9) provides a formula for the allowed energies, since k is a function of E. A zero-value in either of the Kronecker deltas would lead to $k \equiv 0$ (and hence $E \equiv 0$) and hence non-bound motions for the substrate molecules in the two wells, which accordingly results in a failure of the catalytic tandem system (as is apparent the case for these control reactors, which lack necessary 'imprints' and hence accommodations for the formation of tandem catalysis). The catalytic tandem system becomes feasible at MIP-Au-NP-BNPC with

$$
e^{-kb} = \pm\left(1 - \frac{k}{\Omega}\right). \qquad (10)
$$

To solve for E, it pays to adopt some nicer notation. Let

$$X \equiv kb = b\sqrt{-\frac{2mE}{\hbar^2}} \text{ and } \sigma \equiv \frac{1}{b\Omega}, \qquad (11)$$

so that Eqn (10) reads

$$\begin{aligned} e^{-X} &= \pm(1-\sigma X) \\ &= y^{\pm}(X) \end{aligned}. \qquad (12)$$

This is a transcendental equation for X (and hence for E). It can be solved graphically by plotting e^{-X} and $\pm(1-\sigma X)$ on the same grid, and looking for points of intersection (**Scheme S2**). The intersection points are either one or two, depending on the magnitude of σ (more or less than 1). As in the present case, the "imprints" of BNPC allowed accommodations for the hydrolysis of BNPC and the encapsulated Au nanoparticles in the presence of admissible access were responsible for the reduction of NP. It is therefore unrealistic to have the different-type reactions subject to the identical energies in the two functionally-different wells (graphically corresponding to the single intersection-point in **Scheme S2b**). Hence, the catalytic tandem system becomes feasible only with $\sigma<1$. As such, the two intersection-points in **Scheme S2a** show pre-requisites for the natural formation of tandem catalytic-ability at MIP-Au-NP-BNPC (*i.e.*, segregated quantum confinements necessary for the natural formation of tandem catalytic-ability)

$$E_n = -\frac{\hbar^2 X_n^2}{2b^2 m_n} \; (n=1,2). \qquad (12)$$

The segregated quantum confinements in this reactor catalytically constitute two quantum interaction-segregated domains, which lead to the occurrence of tandem catalysis. Hence, once the initial substrate molecules are mounted in this reactor, the substrate molecules would go through the corresponding quantum confinements in the two domains in which one is responsible for the precursor reaction and another one would permit the following reaction. Unlike at reported functional reactors that run tandem catalysis by largely depending on the precise control and man-made isolation of different-type catalytic sites, the tandem catalysis at this reactor run naturally with the segregated quantum confinements which do not involve the precise control and man-made isolation of different-type catalytic sites. In this way, this reactor demonstrates the tandem catalytic-ability.

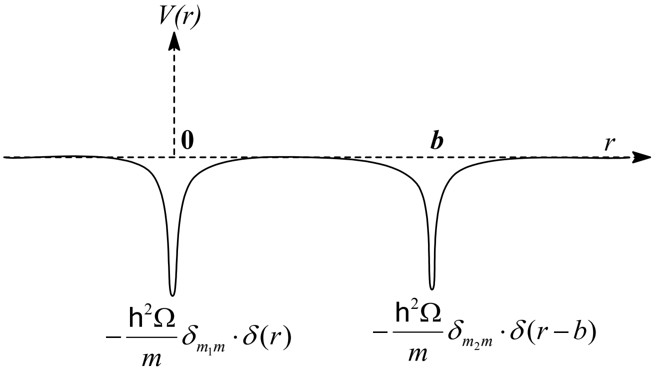

Scheme S1. Suggested tandem δ potential-wells

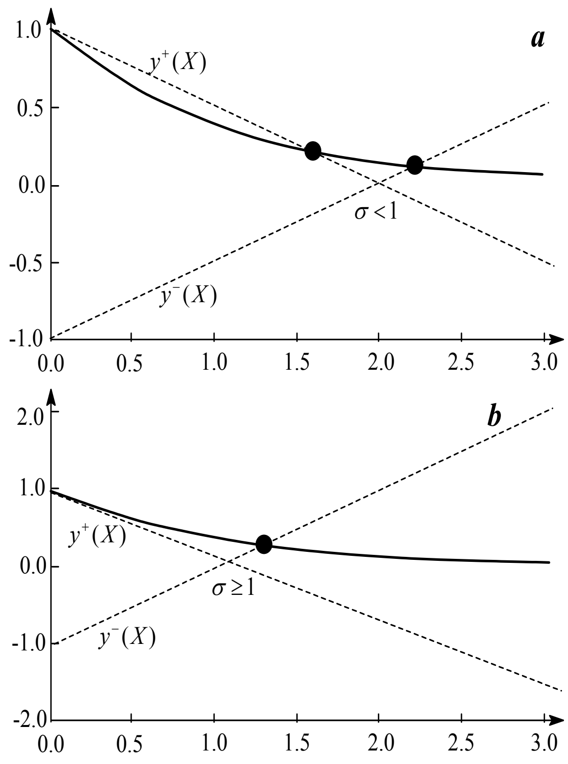

Scheme S2. Schematically graphic solutions to Eqn (12)

References

1 R. J. L. Roy, *J. Quant. Spectr. Rad. Transfer*, 2017, 186, 167-178.

2 X. Antoine, E. Lorin and A. D. Bandrauk, *J. Sci. Comput.*, 2015, 64, 620-646.

3 K. Wang, Z. Jia, X. Yang, L. Wang, Y. Gu and B. Tana, *J. Catal.*, 2017, 348, 168-176.

4 M. Filice and J. M. Palomo, *ACS Catal.*, 2014, 4, 1588-1598.

5 C. Zuo, W. Wei, Q. Zhou, S. Wu and S. Li, *ChemistrySelect*, 2017, 2, 6149-6153.

6 A. Corichi, T. Vukašinac and J. A. Zapata, *Phys. Rev. D*, 2007, 76, 044016.1-044016.16.
